# Knowledge, practice and associated factors of essential newborn care at home among mothers in Gulomekada District, Eastern Tigray, Ethiopia, 2014

**DOI:** 10.1186/s12884-016-0931-y

**Published:** 2016-06-21

**Authors:** Haftom Gebrehiwot Misgna, Haftu Berhe Gebru, Mulugeta Molla Birhanu

**Affiliations:** Department of Nursing, College of Health Sciences, Mekelle University , Mekelle, Ethiopia

**Keywords:** Knowledge, Practice, Essential newborn care

## Abstract

**Background:**

Around the world, more than three million newborns die in their first months of life every year. In Ethiopia during the last five years period; neonatal mortality is 37 deaths per 1000 live births. Even though there is an improvement compared to the past five years, there is still high home delivery 90 %, and high neonatal mortality about the Millennium Development Goal, which aims to be less than 32/1000 live births in Ethiopia. The purpose of this study is to assess maternal knowledge, practice and associated factors of essential newborn care at home in Gulomekada District Eastern Tigray, Ethiopia.

**Methods:**

A community-based cross-sectional study is conducted in 296 mothers from Gulomekada District by using simple random sampling technique. Data entry and analysis is carried out by using Statistical Package for Social Sciences-20. The magnitude of the association between different variables about the outcome variable is measured by odds ratio with 95 % confidence interval. A binary logistic regression analysis is made to obtain odds ratio and the confidence interval of statistical associations. The goodness of fit had tested by Hosmer-Lemeshow statistic and all variables with *P*-value greater than 0.05 are fitted to the multivariate model. Variables with *P* < 0.2 in the bivariate analysis are included in the final model, and statistical significance is declared at *P* < 0.05.

**Result:**

Eighty percent (80.4 %) study participants had good knowledge on essential new born care and 92.9 % had the good practice of essential new born care. About 60 % of mothers applied butter or oil on the cord stump for their last baby. Marital status and education are significantly associated with knowledge, whereas urban residence mothers with good knowledge on essential newborn care and employed mothers are significantly associated with mothers’ practice of essential newborn care.

**Conclusion:**

Almost all mothers know and practice essential newborn care correctly except oil or butter application to the cord stump is highly practiced which should be avoided. Only marital status and educational status are significantly associated with mothers’ knowledge.

**Electronic supplementary material:**

The online version of this article (doi:10.1186/s12884-016-0931-y) contains supplementary material, which is available to authorized users.

## Background

More than half of the approximately 7.5 million neonatal deaths in the world occur in the first four weeks after birth. Ninety-eight percent of these neonatal deaths occur in developing regions, 28 % in the least developed countries. Overall, there were 30 neonatal deaths per 1000 live births; 5 per 1000 in developed and 33 per 1000 in developing regions, and 42 per 1000 in the least developed countries*.* This means that in developing regions, the risk of death in the neonatal period is more than six times than that of developed countries; in the least developed countries, it is more than eight times higher [1].

The proportion of child deaths that occurs in the neonatal period is increasing, and the Millennium Development Goal (MDG) for child survival cannot be met without substantial reduction in neonatal mortality. One of the targets of the MDG is a two-third reduction in infant and child mortality by 2015, it may achieved by upgrading the proportion of births attended by skilled health personnel, increasing immunization against the six vaccine preventable diseases, and improving the status of women through education and enhancing their participation in the labor force [2].

The world has made substantial progress, reducing the under-five mortality rate 41 %, from 87 deaths per 1000 live births in 1990 to 51 in 2011. However, this progress has not been enough, and the target risks being missed at the global level. The global under-five mortality rate needs to be reduced to 29 deaths per 1000 live births—which implies an annual rate of reduction of 14.2 % for 2011–2015, much higher than the 2.5 % achieved over 1990–2011 [3].

Globally, the main direct causes of neonatal deaths are estimated to be preterm birth (28 %), severe infections (26 %), and asphyxia (23 %). Maternal complications in labor carry a high risk of neonatal death, and poverty is strongly associated with an increased risk. Preventing deaths in newborn babies has not been a focus of child survival or safe motherhood programs [4]. While we neglect these challenges, 450 newborn children die every hour, mainly from preventable causes, which is unconscionable in the 21^st^ century [4].

Over a million African babies are estimated to die in the first four weeks of life, but most die at home, uncounted, and invisible to national and regional policies and programs. Global estimates suggest that over two-thirds of newborns could be saved through existing maternal and child health programs [5].

### Statement of the problem

Around the world, more than three million newborns die in their first month of life every year. Many of these deaths can be prevented using proven and cost-effective interventions. Home-Based Newborn Care (HBNC) packages successfully address the leading causes of newborn deaths, decreasing newborn death rates by up to 70 %. Village health workers deliver HBNC (maternal and child health care provided by health extension workers at home level) to vulnerable families at the community level, effectively reaching families that lack access to medical facilities [6].

With 41 neonatal deaths per 1000 live births, the risk of neonatal death is highest in Africa, mainly in the Sub-Saharan regions of Western, Middle and Eastern Africa that have between 42 and 49 neonatal deaths per 1000 live births, whereas Southern and Northern Africa have lower rates. Asia has an average neonatal mortality rate of over 32 per 1000 live births. The highest number of neonatal deaths occurs in Asia, which is where most children are born. As mortality is very high in the South-central Asia sub-region, over 40 % of global neonatal deaths take place here, representing a formidable challenge. Most deaths in the neonatal period occur in the first few days after birth. Consequently, early neonatal mortality represents about 75 % of neonatal mortality, and this applies to all regions of the world. Early neonatal deaths (a death within the first one week after birth) often have obstetric origins [1].

In Ethiopia during the past five-year period, neonatal mortality estimated 37 deaths per 1000 live births and 22 deaths per 1000 live births for post-neonatal mortality. Even though there is improvement about the past five years, there is still high home delivery 90 %, and high neonatal mortality, 37 per 1000 live birth compared to the MDG plan that aimed to be 32 in Ethiopia. Thus, further intervention is needed to sustain the improvement in the maternal and neonatal mortality [2].

Community-Based Newborn Care (CBNC) in Ethiopia is a national package that aims to improve newborn survival through the Health Extension Program. This will involve implementing a newborn care package along the continuum of care from pregnancy to post-birth through frontline community workers, including improving sepsis management (care for and treatment of a newborn with a potentially deadly bacterial blood infection). A set of practices that reduces newborn morbidity and mortality has been identified as essential and these include clean cord care (cutting and tying of the umbilical cord with a sterilized instrument and thread), thermal care (drying and wrapping the newborn immediately after delivery and delaying the newborn’s first bath for at least six hours or several days to the reduce hypothermia risk), and initiating breastfeeding within the first hour of birth [7].

### Significance of the study

Initially the study will be used as a baseline for researchers since limited studies are conducted in the region. In addition, the results of this study are useful to provide an evidence of the gaps found in the area for relevant stakeholders.

### Objectives

Three main objectives of this study are; first to determine the knowledge of mothers on essential newborn care at home, second to assess the practice of mothers on essential newborn care at home and the third is to identify factors associated with essential newborn care by mothers at home.

## Methods

The study is conducted in Gulomekada District, one of the Districts in Tigray. It is located 914 KM from Addis Ababa and 155 KM from Mekelle, the capital of Tigray region. There are 19 Kebeles with a total population of 99,890, of whom 50,944 (51 %) female; out of these female populations 23,474 (46 %) belongs to the reproductive age group. In the past six months in the District, there are 893 mothers that have an infant less than six months of age. A quantitative community-based cross-sectional study is conducted. Study populations are all mothers with an infant of fewer than six months old from all Kebeles of the District.

All mentally capable mothers (conscious and free from a known psychiatric disorder) with an infant less than six months old and mothers who stayed in the respective Kebeles for the past six months are included while seriously ill (mothers with severe medical problem) are excluded.

The sample size for this study is calculated by single proportion sample size formula, and we have taken a total of 296 study subjects. Samples are selected from all villages using simple random sampling. According to the number of mothers with an infant, less than six months old in each village of the Kebeles samples are proportionally selected.

A semi-structured interview questionnaire is prepared by reviewing different kinds of literature. Data is collected by diploma nurses found in the district after they got trained how they should interview the mothers. Participants are selected randomly and the interview is conducted at their home.

Knowledge and practice of mothers are our dependent variables while Socioeconomic and demographic variables such as age, ethnic group, religion, monthly income, occupation, educational status of mothers, exposure to information and ANC follow-up (maternal health facility visit during pregnancy for at least four times) are independent variables for this study.

To ensure data quality questionnaires are prepared in English language and they are translated to the local language of the study area, Tigrigna by experts. Questionnaires are pre-tested in a local area with population having similar socio-demographic status. After pretest had been conducted, there is a correction of questionnaires that are not clear.

Data is entered, analyzed using SPSS-20 and summarized by using tables and figures. Descriptive statistics is computed to determine the knowledge, practice and associated factors of new born care. The magnitude of the association between the different variables about the outcome variable is measured by odds ratio with 95 % confidence interval. Binary logistic regression analysis is made to obtain odds ratio and the confidence interval of statistical associations. The assumptions for binary logistic regression are checked. The goodness of fit had tested by Hosmer-Lemeshow statistic and all variables with *P*-value greater than 0.05 are fitted to the multivariate model. The strength of statistical association is measured by adjusted odds ratio and 95 % confidence intervals. Variables with *P* < 0.2 in the bivariate analysis are included in the final model of multivariate analysis, and statistical significance is declared at *P* < 0.05.

The study is conducted after getting ethical clearance from Mekelle University, College of Health Sciences Ethical Review Committee. Written informed consent is obtained from study participants. They are also adequately informed the purpose of the study and the importance of their participation to confirm willingness of involvement. During processing and analysis of the data, strict confidentiality is maintained.

## Result

### Socio-demographic characteristics of mothers

A total of 296 mothers are included in the study and 100 % of them participated in the interviewed questionnaires (Additional file 1). The mean age of the study subjects and their infants is 30.6 (±6.5 SD) years and 3.3 months (±1.7 SD) respectively. All study participants are from a Tigray region and followed orthodox Christianity. Two hundred fifty three (85.5 %) mothers used health professionals as a source of information about maternal and child health (Table 1).

**Table 1:** Socio-demographic characteristics of mothers’ in Gulomekada District Eastern Tigray Ethiopia May, 2014 (*n* = 296)

Variable and response	Frequency(f)	Percent (%)
Sex of the infant (*n* = 296)		
Male	160	54.1
Female	136	45.9
Educational status (*n* = 296)		
Able to read and write	117	39.5
Unable to read and write	114	38.5
Elementary school	28	9.5
Secondary school and higher education	37	12.5
Marital Status (*n* = 296)		
Single	23	7.8
Married	249	84.1
Divorced	24	8.1
Occupation (*n* = 296)		
House hold wife	257	86.8
Merchant	19	6.4
Governmental organization	10	3.4
student	10	3.4
ANC follow up (*n* = 296)		
Yes	290	98
No	6	2
Residency (*n* = 296)		
Urban	91	30.7
Rural	205	69.3

#### Knowledge of mothers on essential newborn care at home

**Fig. 1:**
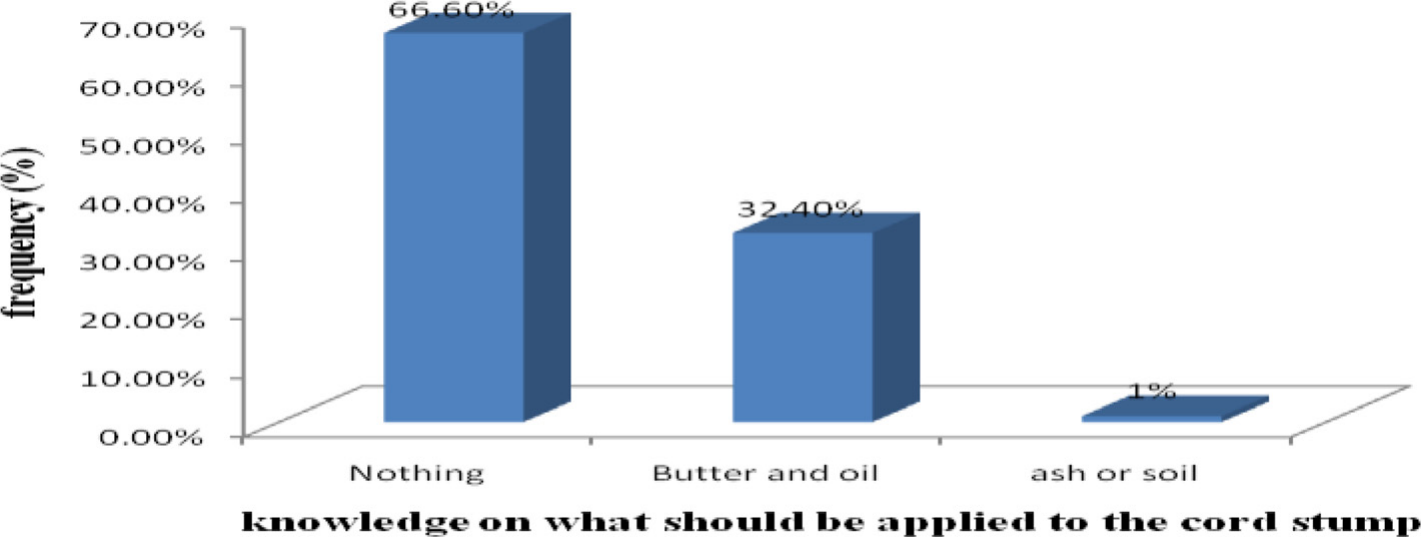
Knowledge of Mothers’ on what should apply to the cord stump in Gulomekada District May, 2014

From the study participants 238(80.4 %) had good knowledge (responded greater than 50 % knowledge questions correctly) on essential newborn care at home, where as 58(19.6 %) of them had inadequate knowledge. Only 66.6 % of the participants responded that nothing should be applied to the cord stump (Fig. 1).

For temperature maintenance of the baby 229(77.4 %) of them answered the baby should be covered with warm cloths and placed on the mother’s abdomen, whereas 15(5.1 %) of them did not know the way of temperature maintenance. Participants are asked about breastfeeding initiation and 238(80.4 %) of them answered the baby should be started breastfeeding immediately and within one hour after birth. Two hundred twenty nine (77.4 %) of them responded the baby should be bathed after 24 h of delivery whereas 9(3 %) of them did not know the exact time for first bathing. Study subjects are asked to their knowledge of newborn danger signs using seven newborn danger signs(Poor sucking or unable to breast feed, Fast breathing, Severe chest in drawing, Hypothermia, Fever, Difficulty in movements or lethargy/unconsciousness, severe umbilical infection, redness of skin around the cord and foul smelling discharge) and 148(50 %) of them stated three and below. Of the 296 study participants 283(95.6 %) responded new tie should be used for cord binding while 7(2.4 %) of them did not know (Table 2).

**Table 2:** Mothers’ knowledge on essential newborn care at home in Gulomekada District Eastern Tigray Ethiopia May, 2014

Variable	Frequency	Percent (%)
Knowledge on breast feeding initiation time (*n* = 296)		
immediately after birth	138	46.6
within one hour after birth	100	33.8
after placenta removed	58	19.6
Knowledge on first bathing time (*n* = 296)		
immediately after birth	37	12.5
after 24 h of delivery	229	77.4
before 24 h of delivery	21	7.1
do not know	9	3.0
Knowledge on what should be the cord tie (*n* = 296)		
new tie	283	95.6
old tie	6	2.0
do not know	7	2.4

#### Practice of mothers on essential newborn care at home

Two hundred seventy-five (93 %) study subjects had a good practice (responded greater than 50 % practice questions correctly) on essential newborn care. Participants are asked about the correct practices on cord cutting, cord tying, application on the cord stump, breast feeding initiation, colostrums feeding, first bath time, immunization and place of delivery.

**Fig. 2:**
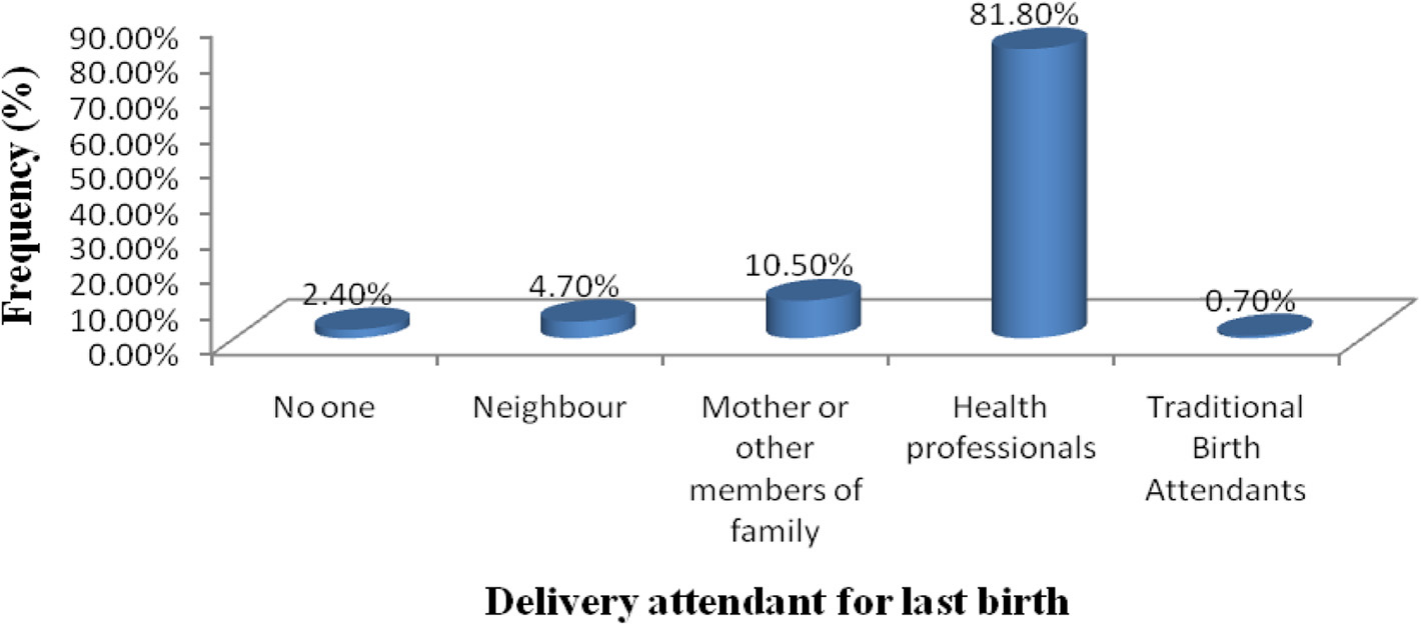
Responses of mothers for delivery attendant of their last baby in Gulomekada District May, 2014

All mothers are asked for the delivery attendant of their last baby and result showed that only 242(81.8 %) are attended by a health professional (Fig. 2). Two hundred eighty (94.6 %) of them responded the cord cutting instrument used is a new blade, and only 16(5.4 %) did not know what they had used for cord cutting. Only 128 (43.2 %) responded nothing is placed under the cord while 131(44.3 %) of them did not know. In their last baby 237(80.1 %), mothers used new cord tie and 7(2.4 %) of them used the unsterilized old tie (Table 3).

**Table 3:** Mothers practice of essential new born care at home in Gulomekada District Eastern Tigray Ethiopia May, 2014

Variable	Frequency	Percent (%)
Cord cutting instrument for your last baby (*n* = 296)		
New blade	280	94.6
do not know	16	5.4
Boiled cord cutting instrument for your last baby (*n* = 296)		
yes	253	85.5
no	15	5.1
do not know	28	9.5
Cord tying instrument for your last baby (*n* = 296)		
new cord tie	237	80.1
boiled cord tie	52	17.6
un boiled cord tie	7	2.3
The material applied to the cord stump (*n* = 176)		
oil or butter	176	100
The status f the towel used for wrapping the baby (*n* = 293)		
clean and dry	262	89.4
new towel	18	6.1
	13	4.4
Baby placed on before placenta out (*n* = 296)		
Ground	39	13.2
mother’s abdomen	181	61.1
with other person	70	23.6
do not know	6	2.0

**Fig. 3:**
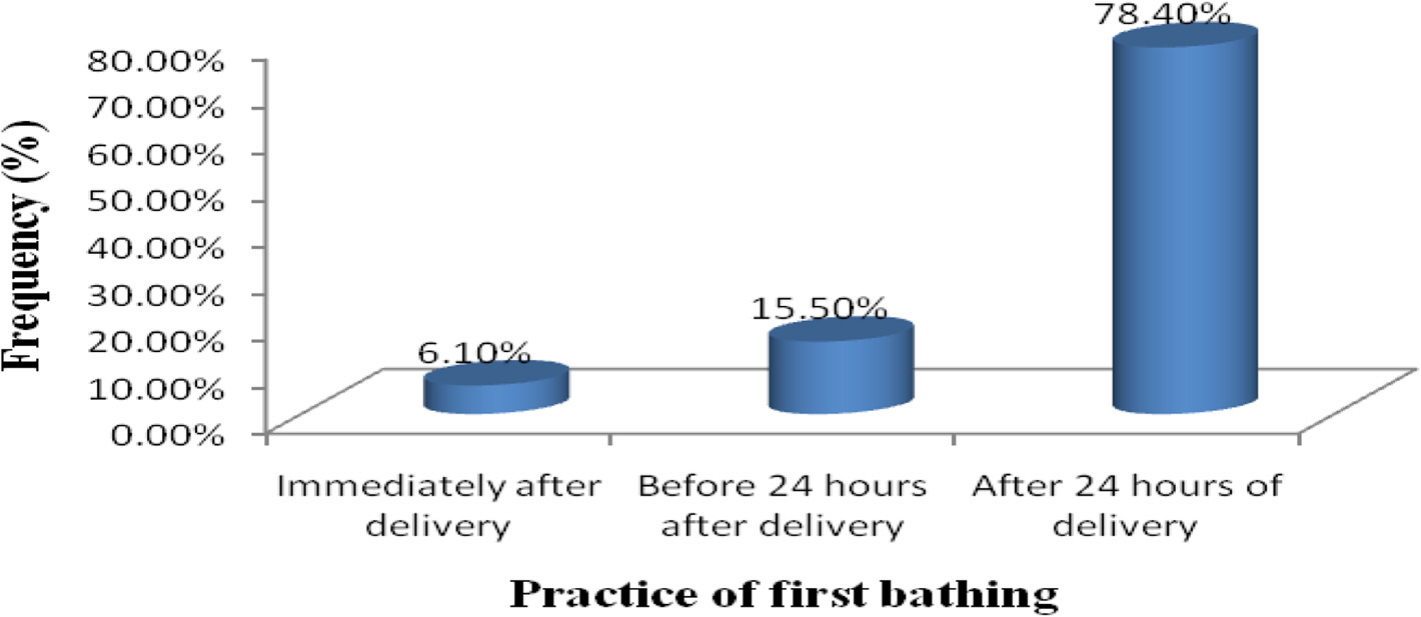
Mothers’ Practice of first time bathing for their last baby in Gulomekada District May, 2014

About 60 % of mothers applied butter on the cord stump for their last baby. Almost all 99 % of babies are placed on mother’s abdomen and wrapped by a towel. The status of towel used for wrapping is new, clean and dry in 280(95.5 %) of participants. Participants also responded to the time of first bathing for their last baby and 232(78.4 %) of them bath their last baby after 24 h of delivery (Fig. 3).

Two hundred ninety-one (98.3 %) mothers feed colostrums for their final baby and the rest of them are not feed colostrums due to carelessness to its advantage. Time for initiation of breastfeeding in their last baby is within one hour of delivery in 276(93.2 %). Majority 290(98 %) of mothers reported their last baby is immunized, and 2 % of them did not immunized due to lack of medication (Table 3).

#### Factors associated with knowledge and practice of mothers on essential newborn care at home level

Assessment of knowledge and practice of essential newborn care of mothers are made by a logistic regression model with the assumption that it helps to predict the extent by which knowledge and practice of essential newborn care could be explained by independent variables.

Only marital status and those able to read and write are significantly associated with knowledge of essential newborn care, the odds of knowing about essential newborn care is 3.0 among married women compared to single, divorced or widowed mothers [OR (95 % CI); 3.0(1.34, 6.76)] whereas the odds of knowing about essential newborn care is 0.34 among mothers who are able to read and write compared to mothers unable to read and write [OR (95 % CI); 0.34(0.16, 0.71)]. Number of live birth [OR (95 % CI); 1.10(0.49, 2.45)], ANC follow up [OR (95 % CI); 2.73(0.44, 17.0)] and occupational status [OR (95 % CI); 2.22(0.57, 8.66) had not significant association with knowledge of mothers on essential new born care (Table 4).

**Table 4:** Factors associated with mothers’ practice of essential newborn care at home in Gulomekada Woreda May, 2014

Variable	Level of Knowledge		OR(95 % CI)	
	Poor	Good	COR	AOR
Number of live birth				
Four and less than 4	34	173	1.88(1.03,3.41)	1.10(0.49,2.45)
> 4	24	65	+1	
ANC follow up				
Yes	55	235	4.27(0.84, 21.7)	2.73(0.44,17)
No	3	3	+1	
Educational status				
Unable read and write	14	102	+1	
Able read write	37	79	0.29(0.15,0.58)	0.34(0.16, 0.71)
Elementary and above	7	57	1.12(0.42, 2.93)	0.78(0.26, 2.32)
Marital status				
Married	43	206	2.24(1.12, 4.50)	3.0(1.34, 6.76)
Single, divorced	15	32	+1	

Multivariable analysis of independent variables shows that place of residence, occupational status and knowledge status of mothers are significantly associated with mothers’ practice of essential new born care. Odds of practicing essential newborn care is 6.57 among urban resident mothers compared to rural resident mothers [OR (95 % CI) 6.57; (1.43, 30.26)], whereas the odds of practicing essential newborn care is 9.97 among employed mothers compared to unemployed mothers [OR (95 % CI) 9.97; (2.57, 38.6)]. Odds of practice is also 5.63 among mothers with good knowledge compared to mothers with poor knowledge [OR (95 % CI) 5.63; (1.99, 15.89)]. Educational status, monthly income, the source of information, mothers’ age, ANC follow-up, marital status and number of live birth had no significant association with the practice of essential newborn care (Table 5).

**Table 5:** Factors associated with mothers’ practice of essential newborn care at home in Gulomekada District Eastern Tigray Ethiopia May, 2014

Variable	Level of practice		OR(95 % CI)	
	Poor	Good	COR	AOR
ANC follow up				
Yes	19	271	7.13(1.22, 41.45)	5.25(0.64, 42.8)
No	2	4	+1	
Occupation				
Unemployed	16	251	+1	9.97(2.57, 38.63)
Employed	5	24	0.30(0.10, 0.90)	
Marital status				
Married	14	235	2.94(1.12, 7.73)	2.65(0.89, 7.91)
Single and divorced	7	40	+1	
Place of residence				
Urban	3	88	2.82(0.81, 9.83)	6.57(1.43, 30.26)
Rural	18	187	+1	
Level of knowledge				
Poor	11	47	+1	5.63(1.99, 15.89)
Good	10	228	5.33(2.14, 13.3)	

## Discussion

The overall knowledge (80.4 %) and practice (92.9 %) of mothers on essential newborn care is good which is higher than an Indian study in which only 76.7 % good knowledge and 66.7 % good practice. Reasons for these differences may range from the cultural background of the mothers to access to health facility and health professionals [8].

Marital status and educational status of mothers had significantly associated with mothers’ knowledge of essential newborn care. The odds of knowing about essential newborn care among married women are 3.0 and the odds of knowing about ENBC among those who are able to read and write is 0.34. But there is no association with marital status in a similar study conducted in Sri Lanka which shows strong association with employment and ANC follow up. This difference might be due to married women got an advising support from their husbands [9].

Mothers who had good knowledge are significantly associated with the practice of essential newborn care with the odds of 5.63. This is in-line with a study conducted in Nepal in which mothers who knew about essential newborn care are more likely to practice it. In our study, there is no significant association between educational status and essential newborn care practice, however, there is a significant association in a similar study conducted in Nepal and Bangladesh, which shows highly educated mothers are more likely to practice essential newborn care because they might be learnt what should be the ENBC practice. Differences regarding educational status may be caused by the health extension program of the country that provides adequate service for all mothers regardless of educational status [10, 11].

Urban resident mothers are also significantly associated with practice of essential newborn care having the odds of 6.57 and the odds of employed mothers on essential newborn care practice are 9.97 which is in line with a study conducted in Sri Lanka that employed mothers are significantly associated with practice [9].

In our study education and income had not significant association with the practice of essential newborn care in contrast to a study conducted in Nigeria and Bangladesh that had a significant association. There is also no association with practice of essential newborn care and, ANC follow-up and marital status in this study. The difference between these studies may be due to HEWs (Professionals maternal and child health care providers at home level) house to house visit to all mothers regardless of educational status and income [12, 13].

## Conclusions

The finding about essential newborn care knowledge and practice of mothers revealed that 80.4 % had good knowledge and 92.9 % had good practice. Most mothers had good knowledge on temperature maintenance, breast feeding initiation and first bathing time. In addition to their knowledge, almost all mothers practiced the main essential newborn care except a substance (oil and butter) application to the cord stump. The majority of mothers apply oil and butter on the cord stump which may lead to many neonatal infections. However, mothers should not apply any preparations on the cord. Ninety-eight (98 %) Mothers had ANC follow-up though 18.2 % mothers are still delivered at home.

In general, this study tried to identify the factors associated with knowledge and practice of essential newborn care at home level and only marital status and educational status are significantly associated with mothers’ knowledge of essential newborn care. Similarly, Knowledge of essential newborn care, place of residence and occupation are significantly associated with mothers’ practices of essential newborn care.

The limitation of the study may be recall bias of respondents and the study design, being cross sectional. These limitations may affect the result negatively by mothers’ failure to remember what they do for their children and the study design also can not show real cause and effect association. The other limitation of this study is also the imbalance between mothers’ knowledge and practice of unhygienic substance application on the cord stump which should be addressed.

Based on the findings from this study the following recommendations are made:

- The mothers should get continuous health education on this issue of newborn care by health care providers specially Health Extension Workers and by the community women development army.
- Regional health offices and their respective zonal and districts should provide short-term training on newborn care especially to HEW (health care service providers at home level), WDA (a network of one to five mothers in the community), and even community leaders.
- Further researches should be conducted on areas of essential newborn care to identify more gaps.

## Abbreviations

ANC, antenatal care; CBNC, community based newborn care; CHDK, clean home delivery kit; EDHS, Ethiopian demographic health survey; HBNC, home-based newborn care; HEW, health extension workers; MDG, millennium development goal; SPSS, statistical package for social science; UNICEF, United Nations children’s fund; WDA, women development army; WHO, World Health Organization

## Additional file

Additional file 1: English Version Questionnaires. (PDF 212 kb)
