# Supplementary material for: Knowledge, practice and associated factors of essential newborn care at home among mothers in Gulomekada District, Eastern Tigray, Ethiopia, 2014
Source: BMC Pregnancy Childbirth. 2016 Jun 21;16:144. doi: 10.1186/s12884-016-0931-y (PMC4915039; doi:10.1186/s12884-016-0931-y)
Supplement: Additional file 1: — English Version Questionnaires. (PDF 212 kb) [file 12884_2016_931_MOESM1_ESM.pdf]

English Version Questionnaires

Part-I. Socio-demographic characteristics of mothers with a child less than 6 month age.

**Nº**	**Question**	**Response**	**Skip**
100	Age of the mother	_____	
101	Age of the child	______	
102	Sex of the child	1. Male 2. Female	
103	Religion	1. Orthodox<br>2. Muslim<br>3. Protestant<br>4. Catholic<br>5. Other________	
104	Ethnicity	1. Tigray 2. Other_____	
105	Educational back ground	1. Not able to read and write<br>2. Primary school<br>3. Secondary school<br>4. Diploma and above	
106	Marital status	1. Single<br>2. Married<br>3. Widow<br>4. Divorced	
107	Occupation	1. House wife<br>2. Merchant<br>3. Government employee<br>4. Non government employed<br>5. Student<br>6. Other____	
108	Monthly income(ETB)	1. <500<br>2. 500-1000<br>3. >1000<br>4. Do not know	
109	Number of children born alive	______	
110	Have you visited/counseled for ANC in your last pregnancy and delivery?	1. Yes 2. No	
111	Place of residency	1. Urban 2. Rural	
112	Have source of information that you follow about maternal and child health?	1. Yes<br>2. No	If "no" leave question number 113
113	What are the sources of information you have?	1. Radio, Television<br>2. Health professional<br>3. Community leaders	

Part II. Knowledge questions regarding essential newborn care for mothers with an infant less than 6 month old.

Nº	Questions	Response	Skip
200	What should be applied on the umbilical stump after it is cut?	1. Leave as it is<br>2. Apply butter or oil<br>3. Apply anti biotic cream<br>4. Apply ash or soil	
201	How can you maintain the normal body temperature of the baby?	1. using warm cloths<br>2. kangaroo method<br>3. warm cloth and keeping the baby close to the mothers body<br>4. Do not know	
202	When you should start the first breast feeding?	1. Immediately after birth<br>2. Within one hour after delivery<br>3. After the placenta is removed	
203	When you should to have first bathing for the baby after delivery?	1. Immediately after delivery<br>2. Before 24 hour of delivery<br>3. After 24 hour of delivery<br>4. I do not know about the exact time for bathing	
204	What danger signs of a new born did you know?	1. Poor suckling or unable to breast feed<br>2. Fast breathing<br>3. Severe chest in drawing<br>4. Hypothermia<br>5. Fever<br>6. Difficulty in movements or lethargic/unconsciousness<br>7. severe umbilical infection, redness of skin around the cord and foul smelling discharge	
205	What material should be used for tying the cord after it is cut?	1. New string/thread<br>2. Old string<br>3. Fiber from local plant<br>4. Cord should not tied<br>5. I do not know	

Part-III. Practice questions regarding essential newborn care for mothers with an infant less than 6 month old.

Nº	Questions	Response	Skip
300	Who assisted or attended to you during delivery?	1. no attendant<br>2. neighbor<br>3. mother<br>4. other family member<br>5. Nurse or midwife<br>6. Health extension worker<br>7. Traditional birth attendant<br>8. others ____	
301	What instrument was used to cut the cord?	1. New blade<br>2. Old blade<br>3. Household knife<br>4. Unknown<br>5. Others __	
302	Did the instrument boiled before used?	1. Yes<br>2. No<br>3. Do not know	
303	What was used to tie the cord?	1. New tie<br>2. Boiled string or thread<br>3. Un boiled string<br>4. Do not know<br>5. Other___	
304	On what surface was the cord cut?	1. Plastic disc<br>2. Metal coin<br>3. Wood<br>4. Nothing<br>5. Other___<br>6. Do not know	
305	Did the person who handled the baby assisting with delivery washed hands with soap and water first?	1. Yes<br>2. No<br>3. Do not know	
306	Did anybody apply anything on the stump after the cord was cut?	1. Yes<br>2. No<br>3. Do not know	If "no" or "I do not know" skip question 24
307	What did you applied?	1. Oil or butter<br>2. Ash<br>3. Anti biotic cream<br>4. Ointment/powder<br>5. Other___<br>6. Do not know	
308	Was your baby wrapped in cloth or put on mother's body and covered with cloth before the placenta was delivered?	1. Yes<br>2. No<br>3. Do not know	If "no" or "I do not know" skip question 26

309	What was the condition of the cloth for wrapping?	1. Clean cloth<br>2. Dry cloth<br>3. New cloth<br>4. Used cloth<br>5. Wet cloth<br>6. Other____<br>7. Do not know	
310	When did you bath the baby after birth?	1. Immediately after birth<br>2. Before 24 hours<br>3. After 24 hours<br>4. Do not know	
311	Where was the baby placed before placenta was delivered?	1. Floor<br>2. Besides the mother’s body<br>3. With someone else<br>4. Other___<br>5. Do not know	
312	Did you give the baby colostrums the first liquid that comes from your breast?	1. Yes<br>2. No<br>3. Do not know	If “yes” skip question 30
313	Why you did not give colostrums?	1. It is harmful for the baby<br>2. Prohibited by elderly<br>3. Due to ignorance to its advantage<br>4. Do not know	
314	What was the baby fed on first?	1. Breast milk/colostrums<br>2. Breast milk from other woman<br>3. Formula feed-cow’s milk<br>4.Sugr water<br>5.Plain water<br>6.Honey<br>8. Others _____	
315	How soon after birth was the baby breastfed?	1. within the first hour after delivery<br>2. After one hour<br>3. Do not know	
316	Did you start immunization?	1. Yes<br>2. No	If yes skip question 34
317	If no why you did not immunized the baby?	1. Family members did not allow me.<br>2. Baby was sick<br>3. Baby was weak<br>4. Distance to health facility is far<br>5. Health professionals were not cooperative<br>6. Lack of necessary logistics in the health facility.<br>7. Do not know	
318	Where did you deliver you last child?	1. Home by the help of HEW<br>2. Home without help<br>3. Health post<br>4. Health center<br>5. Hospital	If you deliver at home proceed to question 319
319	Why did you deliver at home?	1. Preference for home delivery<br>2. Home delivery is easy and convenient<br>3. All my previous deliveries were at home	

4. Onset of labor before the expected date
5. Lack of transport during labor and Hospital is too far
6. Family members prefer home delivery
7. Fear of hospital-precipitate labor
8. Others -------
